# Boron doping-induced interconnected assembly approach for mesoporous silicon oxycarbide architecture

**DOI:** 10.1093/nsr/nwaa152

**Published:** 2020-07-02

**Authors:** Guanjia Zhu, Rui Guo, Wei Luo, Hua Kun Liu, Wan Jiang, Shi Xue Dou, Jianping Yang

**Affiliations:** State Key Laboratory for Modification of Chemical Fibers and Polymer Materials, International Joint Laboratory for Advanced Fiber and Low-dimension Materials, College of Materials Science and Engineering, Donghua University, Shanghai 201620, China; State Key Laboratory for Modification of Chemical Fibers and Polymer Materials, International Joint Laboratory for Advanced Fiber and Low-dimension Materials, College of Materials Science and Engineering, Donghua University, Shanghai 201620, China; State Key Laboratory for Modification of Chemical Fibers and Polymer Materials, International Joint Laboratory for Advanced Fiber and Low-dimension Materials, College of Materials Science and Engineering, Donghua University, Shanghai 201620, China; Institute for Superconducting & Electronic Materials, Australian Institute of Innovative Materials, University of Wollongong, North Wollongong, NSW 2500, Australia; State Key Laboratory for Modification of Chemical Fibers and Polymer Materials, International Joint Laboratory for Advanced Fiber and Low-dimension Materials, College of Materials Science and Engineering, Donghua University, Shanghai 201620, China; Institute for Superconducting & Electronic Materials, Australian Institute of Innovative Materials, University of Wollongong, North Wollongong, NSW 2500, Australia; State Key Laboratory for Modification of Chemical Fibers and Polymer Materials, International Joint Laboratory for Advanced Fiber and Low-dimension Materials, College of Materials Science and Engineering, Donghua University, Shanghai 201620, China

**Keywords:** mesoporous materials, boron doping, interconnected assembly, energy storage

## Abstract

Despite desirable progress in various assembly tactics, the main drawback associated with current assemblies is the weak interparticle connections limited by their assembling protocols. Herein, we report a novel boron doping-induced interconnection-assembly approach for fabricating an unprecedented assembly of mesoporous silicon oxycarbide nanospheres, which are derived from periodic mesoporous organosilicas. The as-prepared architecture is composed of interconnected, strongly coupled nanospheres with coarse surfaces. Significantly, through delicate analysis of the as-formed boron doped species, a novel melt-etching and nucleation-growth mechanism is proposed, which offers a new horizon for the developing interconnected assembling technique. Furthermore, such unique strategy shows precise controllability and versatility, endowing the architecture with tunable interconnection size, surface roughness and switchable primary nanoparticles. Impressively, this interconnected assembly along with tunable surface roughness enables intrinsically dual (both structural and interfacial) stable characteristics, achieving extraordinary long-term cycle life when used as a lithium-ion battery anode.

## Teaser

A boron doping-induced interconnected-assembly approach is presented to construct an unprecedented assembly of boron doped silicon oxycarbide nanospheres, which are derived from periodic mesoporous organosilicas.

## INTRODUCTION

‘Assembly’ describes a kind of hierarchical architecture of primary building blocks, and has widespread applications in various fields such as biological medicine [1,2], catalysis [3], optical electronics [4], energy storage [5,6], and so on [7]. The enhanced collective properties of such building blocks compared with those of isolated nanoparticles have resulted in assembly of nanosized particles into micrometer-scale secondary structures emerging as a promising solution to integrate the merits of both nanomaterials and microstructures in development of advanced energy storage materials [8–11]. Specifically, when an assembly is used as an electrode material, the risk of side reactions can be prominently mitigated benefiting from the much lower interfacial area, achieving enhanced overall energy density in batteries [12–14]. Meanwhile, the large interparticle resistance caused by the reduced particle size can also be improved remarkably through dense assembly of small primary nanoparticles [15–17]. Furthermore, the tap density of a micrometer-sized assembly results in thinner electrodes and shorter electron pathways at the same mass loading [18].

Assembly protocols reported thus far can generally be categorized as solvent evaporation induced self-assembly [19], microemulsion approach [20], spray drying [21], and other methods such as layer-by-layer assembly [22] and external fields [23]. Self-assembly of monodisperse colloidal nanoparticles induced by solvent evaporation is a complicated process, involving various driving forces and requiring exquisite control over parameters [24]. Emulsion-based assembly and spray drying have been widely adopted to construct secondary microspheres. Of notable success are the pomegranate inspired silicon-based anode materials, which exhibit stable interphase and thus durable cycle life [25,26]. Layer-by-layer assembly relies on electrostatic attraction between oppositely charged species, in which dense assembly is hard to attain [22]. External electric or magnetic field induced assembly of nanoparticles into well-defined structures originates from polarization of primary particles, involving direction-dependent assembly [27]. Despite various assembly tactics, the driving forces based on the aforementioned methods endow weak interactions (e.g. Coulombic, van der Waals and dipolar) between nanoparticles, which unfortunately can bring about structural defects or disruption in certain applications. Targeting this issue, assembly of primary nanoparticles into a robustly interconnected assembly that intrinsically contributes to the interfacial stability of the architecture is highly desirable but remains an enormous challenge.

In this work, we demonstrate a novel boron doping-induced interconnection-assembly (BDIIA) approach to fabricate an unprecedented assembly of boron doped silicon oxycarbide nanospheres (B-SiOC) derived from periodic mesoporous organosilicas (PMOs) [28]. The as-formed B-SiOC assembly is composed of interconnected, strongly coupled nanospheres with rough surfaces. We find that the surface component of primary nanoparticles plays an important role in tuning the tough connection between nanospheres. Through detailed analysis of the boron doping species and a series of comparative experiments, a melt-etching and nucleation-growth mechanism is proposed to elucidate the formation process. Such robust assembly of primary nanoparticles is demonstrated to be powerful in addressing the interface instability via densely packed nanoparticles, giving rise to enhanced cycling stability as lithium-ion battery anodes.

## RESULTS AND DISCUSSION

**Figure 1.**
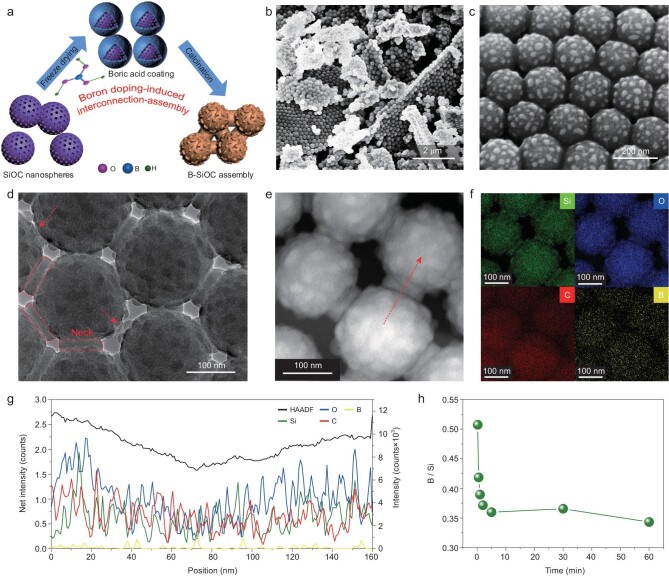
(a) Schematic illustration of the boron doping-induced interconnection-assembly (BDIIA) process. (b, c) FESEM and (d) TEM images of the B-SiOC-3 assembly (inset is the photograph of a neck, a kind of pipe fitting). (e) Dark-field TEM image and (f) the corresponding elemental mapping of the B-SiOC-3 assembly. (g) EDS line mapping profiles along the direction indicated by the red arrow in (e) (red outlined area corresponds to the ‘neck’-like connection location). (h) Dissolving-time-dependent boron concentrations relative to silicon of the B-SiOC-3 sample as measured by ICP, indicating that boron is present on the surface of particle.

BDIIA was achieved by doping boron (B) into SiOC nanoparticles (Fig. 1a). First, an evenly dispersed aqueous solution containing SiOC nanospheres and boric acid was subjected to a freeze-drying process. Subsequently, thermal reduction of boric acid with SiOC nanoparticles in a mixed H_2_/Ar gas was performed, during which a B-SiOC assembly was fabricated. The mass ratios of boric acid to SiOC nanoparticles were set as 1 : 10, 3 : 10, 5 : 10 and 10 : 10, and the products were denoted as B-SiOC-1, B-SiOC-2, B-SiOC-3 and B-SiOC-4, respectively. Figure 1b and c shows the field emission scanning electron microscopy (FESEM) and transmission electron microscopy (TEM) images of the B-SiOC-3 assembly, which exhibits densely packed interparticle connection. Such connection between adjacent nanoparticles was tough (Fig. 1d, indicated by the red-outlined area), looking like a ‘neck.’ Despite some defective connections (Fig. 1d, indicated by red arrows), most of the adjacent nanoparticles coupled strongly with each other. FESEM and TEM images of B-SiOC-3 nanoparticles before calcination show randomly packed nanospheres with smooth surfaces, indicating that assembly occurred at the stage of calcination (Fig. S1). To learn the composition of B-SiOC-3 assembly, dark-field TEM images and corresponding energy dispersive X-ray spectrometer (EDS) elemental mapping were obtained (Fig. 1e and f), from which Si, O, C and B elements coexisted, demonstrating successful doping of B. To confirm the constitutes and distribution of all elements across the ‘neck,’ EDS line elemental dispersion was performed (Fig. 1g), from which Si, O, C and B were found to be distributed evenly in both ‘neck’ and nanoparticle locations, indicating interfacial reconfiguration of SiOC nanoparticles during the doping process. Further, the presence and distribution of B in B-SiOC-3 sample was probed with use of a time-dependent inductively coupled plasma (ICP) test (Fig. 1h), which revealed that the B/Si mass ratio dropped from 0.51 to 0.34 with increase in dissolving time, indicating that B is present on the surface of nanoparticles [29].

**Figure 2.**
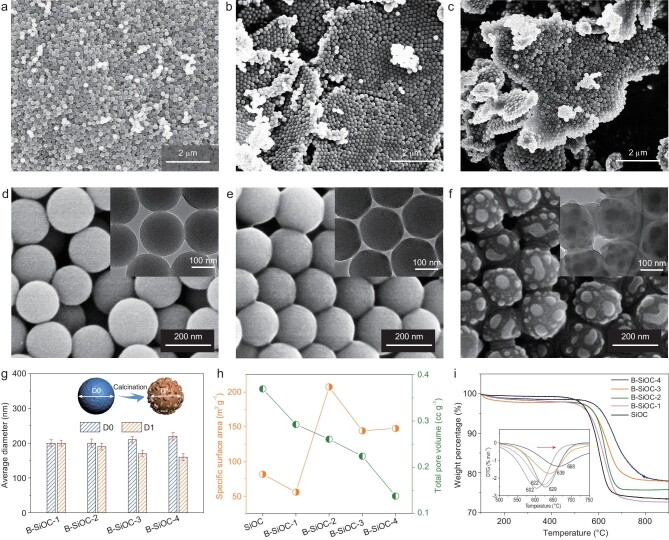
FESEM images of B-SiOC-1 (a, d), B-SiOC-2 (b, e) and B-SiOC-4 (c, f). Insets in (d–f) are the TEM images of the corresponding three samples. (g) Average diameters of B-SiOC samples (D0 and D1 refer to the diameters before and after calcination, respectively). (h) Specific surface area and total pore volumes of the B-SiOC samples. (i) TGA and DTG curves of the B-SiOC samples under an air atmosphere.

Interestingly, it was found that this ‘neck’-like connection could be tuned by varying the mass ratio between boric acid and SiOC nanoparticles. When this ratio was 1 : 10, the as-prepared B-SiOC-1 sample showed a partially narrow ‘neck’ connection and random assembly (Fig. 2a and d). Upon increasing this ratio to 3 : 10, the value of which is slightly lower than that for preparing the B-SiOC-3 sample, overall interparticle assembly occurred, evidenced by the FESEM and TEM images of B-SiOC-2 (Fig. 2b and e). Further increasing the ratio to 10 : 10 could cause excessive growth of the ‘neck’ and highly rough surfaces (Fig. 2c and f). Notably, the assembly process did not occur during lyophilization, which was further certified by FESEM images of samples before calcination (Fig. S2). Moreover, the dark-field TEM imaging and elemental mapping of B-SiOC-4 showed that Si, O, C and B were distributed uniformly, in accordance with B-SiOC-3 (Fig. S3a and b). The elemental dispersion of the rough surface bulges confirmed by EDS line scans further revealed homogeneously distributed Si, O, C and B, suggesting reconstruction of surface morphology when performing B doping (Fig. S3c). To explore the extent of surface morphologic transformation, diameter statistics of the four B-SiOC samples before and after calcination were performed (Fig. 2g). The average diameters of samples before calcination increased from ∼200 nm to ∼220 nm with increasing boric acid content. After calcination, the size of the B-SiOC-1 was almost unchanged, while there was a significant reduction in diameter for the B-SiOC-4 sample, suggesting that depth of surface reconstruction is associated with concentration of boric acid. To evaluate the influence of assembly on the specific surface area of the four samples, Brunauer-Emmett-Teller (BET) nitrogen adsorption was conducted (Fig. S4). With an increasing amount of boric acid in the synthesis system, the total pore volume decreased as expected. However, the specific surface area displayed a distinct trend on account of assembly and surface roughness. More concretely, the specific surface area dropped from 81.58 m^2^ g^−1^ to a relatively low value of 55.77 m^2^ g^−1^ for B-SiOC-1 compared with SiOC without B doping, indicating poor assembling and a relatively smooth surface, whereas the specific surface area of B-SiOC-2 achieved its highest value of 207.43 m^2^ g^−1^. By further increasing the amount of boric acid, the specific surface area declined, indicating that a highly rough surface led to negative contribution to the specific surface area.

Detailed chemical characterization of the B-SiOC samples was further conducted (Fig. S5). Fourier transform infrared (FTIR) spectroscopy was carried out to confirm the B doping (Fig. S5c). The peak centered at 1629 cm^−1^ was assigned to the C=C stretching vibration, and the peak at 1388 cm^−1^ was attributed to the B-O stretching vibration. In addition, the peak emerging at 1112 cm^−1^ was assigned to both Si-O and B-C stretching vibrations, confirming successful doping of B in the SiOC nanoparticles. The X-ray diffraction (XRD) pattern (Fig. S5a) of B-SiOC samples before calcination displayed a combination of a broad peak at around 22°, which was attributed to amorphous SiO_x_ and carbon in SiOC nanoparticles, and well-defined diffraction peaks at 14.7° and 28.2°, which could be indexed to the (010), (002) reflections of boric acid, demonstrating a highly crystalline coating layer. After calcination, the diffraction peaks assigned to boric acid disappeared, indicating thermal reduction of boric acid, and the appearance of a broad peak at around 43° was indexed to the (100) reflections of graphite, suggesting that B doping enhanced the graphitization degree of carbon (Fig. S5b). Raman spectra of the B-SiOC samples were further conducted to confirm the graphitization extent (Fig. S5d). The I_D_/I_G_ ratios were calculated to be 0.95, 1.00, 0.93 and 0.95, for the samples of B-SiOC-1, B-SiOC-2, B-SiOC-3 and B-SiOC-4, respectively, indicating a high degree of graphitization. It could also be expected that B doping would enhance the stability of carbonaceous matrix as the binding energy of the C–B bond is higher than that of the C–C bond [30]. The structural stability of B-SiOC assemblies was measured through thermogravimetric analyses (TGA) in air (Fig. 2i). There was an upward shift of the weight-loss temperature from 602°C to 668°C along with increasing concentration of boric acid.

**Figure 3.**
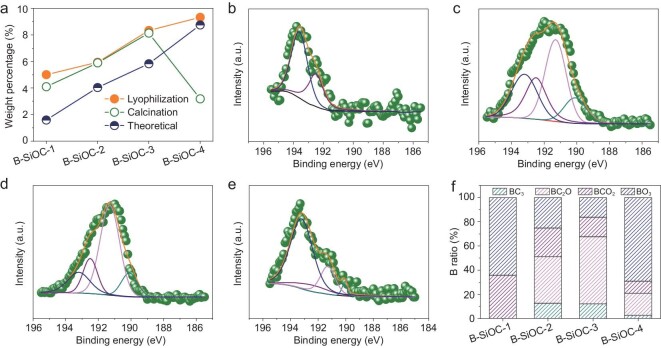
(a) Boron weight percentage estimated from XPS and theoretical values calculated from the quantity added, further indicating surface distribution of boron. B 1s regions of the high-resolution XPS spectra of the B-SiOC-1 (b), B-SiOC-2 (c), B-SiOC-3 (d) and B-SiOC-4 (e). (f) Percentages of different B types in the four samples.

To further identify the chemical bonding environment of B dopant on the surface of the assemblies, X-ray photoelectron spectroscopy (XPS) was conducted (Figs 3 and S6). Generally, the mass percentage of B in these samples before and after calcination showed no obvious change for relatively low contents of boric acid based on the XPS results (Fig. 3a). Increasing the concentration of boric acid resulted in a lower percentage of B, probably because of invalid decomposition of boric acid [31]. Remarkably, the theoretical average percentage of B in the sample was lower than the corresponding values obtained from XPS, further proving surface doping of B. Figure S6b–e presents the C 1s spectra of B-SiOC samples before and after calcination. The sp^2^ carbon peak after calcination become broader relative to that before calcination, suggesting formation of new bonding between carbon and heteroatoms. The deconvoluted high-resolution C 1s spectrum of all B-SiOC samples after calcination was assigned to five C species: C=C at ∼284.2 eV, sp^2^C at ∼284.8 eV, C−O/C−O−B at ∼285.6 eV, C=O at ∼286.5 eV and O−C=O at 289.3 eV, respectively (Fig. S7). Similarly, the high-resolution B 1s spectra of all samples before calcination showed the same peak at ∼193.5 eV, which could be assigned to the B−OH bond in boric acid. After calcination, the deconvoluted B 1s spectrum of all samples gave four individual peaks centered at ∼193.2, 192.5, 191.3 and 190.1 eV, which corresponded to BO_3_, BCO_2_, BC_2_O and BC_3_, respectively (Fig. 3b–e). Typically, the BC_3_ structure is ascribed to substitute of one B atom in graphitic carbon; the BCO_2_ and BC_2_O structures suggest that a B atom replaces the C atom at the edge or defect sites of the carbon framework [32], while the BO_3_ structure indicates doping of B_2_O_3_ with SiO_x_ in SiOC nanoparticles. The ratios of the four types of B-doped structures are summarized in Fig. 3f. Except for B-SiOC-1, all other samples possessed all four types of B-doped structures. Both B-SiOC-1 and B-SiOC-4 samples had significant BO_3_ structure, suggesting preferred B doping with SiO_x_ species rather than with carbon matrix in these two cases. The percentages of the BC_3_ structure in B-SiOC-2 and B-SiOC-3 were 12.6%, and 12.1%, respectively, indicating more heteroatoms doping into the graphitic carbon framework. These distinct boron bonding characters endowed different interface features to the assemblies, which may have different roles in subsequent electrochemical performance.

**Figure 4.**
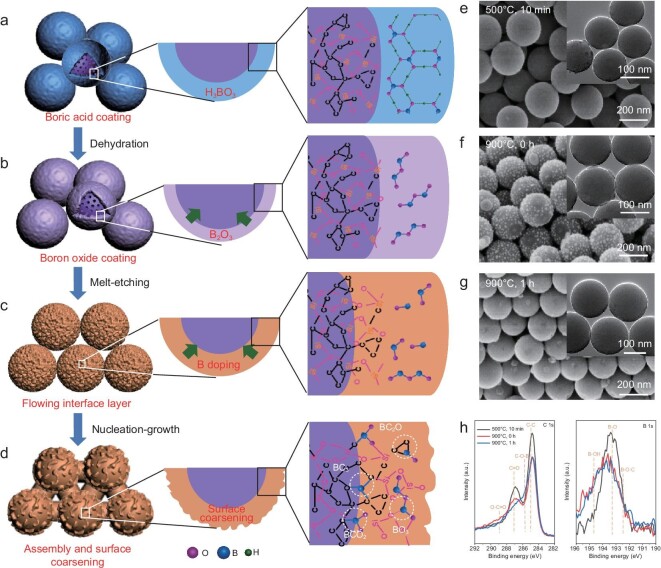
(a–d) Schematic illustration of the assembly formation. (e–g) FESEM images of B-SiOC-2 at different reaction stages, and insets are corresponding TEM images. (h) C 1s and B 1s regions of the high-resolution XPS spectra of the B-SiOC-2 at different reaction stages.

It is well established that high temperature (≥900°C) can trigger a direct solid state reaction between graphitic carbon with boron acid or boron oxide [33]. On the other hand, borosilicate glass phase can be formed when annealing boron oxide and silicon oxide above their glass transition temperature [34]. In the case of SiOC nanoparticles, both carbon and Si−O−Si network exist in molecularly hybrid forms across the whole particle, suggesting that both components can potentially react with boron oxide. Based on this consideration, a melt-etching and nucleation-growth mechanism was proposed (Fig. 4). At the initial stage, crystalline boronic acid coating layers experienced a dehydration process to produce boron oxide at elevated temperature. Subsequently, the solid boron oxide shells become melt when the temperature is above its glass transition temperature (∼253°C) [35]. As the melt structure of boron oxide is complicated and temperature-dependent, especially the inconstant boron-oxygen coordination number, here we draw only its molecular form for simplification (Fig. 4b) [36]. On the principle of binary phase diagram of B_2_O_3_ and SiO_2_, it was reasonable to speculate that a part of B_2_O_3_ can penetrate into SiO_x_ species at the interphase [37,38], leading to a melt phase composed of C, SiO_x_ and B_2_O_3_. This flowing interface layer acts like a solution, which can induce dense packing of the nanoparticles (Fig. 4c). Meanwhile, doping of B into C and SiO_x_ concurrently proceeds. When it starts to cool down, nucleation and recrystallization of these melt regions could cause surface coarsening and an interconnected assembly of B doped SiOC nanoparticles could form (Fig. 4d).

To verify this mechanism, a time-dependent study was conducted by collecting the intermediate products of the B-SiOC-2 sample at different reaction stages. As shown in Fig. 4e, the SiOC nanoparticles still had a random arrangement when the temperature rose to 500°C. When the calcination temperature rose to 900°C without residence time, an interconnected assembly was initiated with rough-surfaced nanoparticles, suggesting that partial etching occurs on the surface of SiOC nanoparticles by molten boron oxide. After reaction for 1 h, the assembly was preserved with a less rough surface, indicating reconstruction of the surface chemical bonding environment as the etching proceeded. The progressive XPS peaks reconfirmed the melt-etching process. With the decomposition of boric acid, obvious C=O species could be discerned from the high-resolution C 1s XPS spectra (Fig. 4h), which can probably be attributed to partial oxidation of amorphous carbon at the interface by boron oxide. As the reaction proceeded, the peak attributed to C=O species became weak and the high-resolution B 1s spectrum became broader, suggesting new bons being formed between carbon and heteroatoms. Simultaneously, the peaks in both high-resolution Si 1s and O 1s XPS spectra shifted to higher binding energies as a result of the increased oxidation state of Si−O−B species, giving further proof to the rebuilding of surface chemical bonding.

To gain more insight into the role played by surface components, a series of comparable experiments was carried out. To be comparable, the mass ratio between boric acid and precursors was set to a fixed value (1 : 2). When pure SiO_2_ nanoparticles were used, a glass-like transparent solid was obtained and the corresponding FESEM images showed an irregular aggregate (Fig. S9), whereas no assembly and surface coarsening were observed when only carbon nanospheres were used as precursor (Fig. S10), indicating that the existence of silicon oxide played a decisive role in driving surface melting and subsequent formation of the interconnected assembly with rough surface. Further, if SiO_2_@carbon core-shell nanospheres were employed as precursors, only random fine nanoparticles decorated the surface of carbon spheres (Fig. S11), which might be caused by incomplete coating. Surprisingly, when other SiOC nanoparticles derived from 1,2-bis(triethoxysilyl) ethenylene (BTEE) were introduced, a partially interconnected assembly with rough surface was observed (Fig. S12). To clarify the doping form of B, high-resolution B 1s XPS was conducted to compare the differences among these samples (Figs S13 and S14). In the case of SiO_2_, there was only one peak assigned to BO_3_, indicating co-fusion between boron oxide and silicon oxide. In the carbon nanospheres, no BO_3_ was observed, while the other three species, BCO_2_, BC_2_O and BC_3_, were confirmed, implying that complete B heteroatoms entered into both edge and inside regions of carbon. However, no BC_3_ was detected for the SiO_2_@carbon core-shell structure, suggesting preferred doping occurred with the silicon oxide component and the defect or edge sites of the carbon layer. For the case of SiOC precursor derived from BTEE, only BO_3_ and BCO_2_ species were obtained, with the absence of the other two doping forms probably a result of the relatively low degree of graphitizing. All these results clearly demonstrate that the component constitute within the interface plays a vital role in conducting formation of the neck-like connection and surface roughening. Moreover, we demonstrated the versatility of this strategy by introducing a PMOs coating layer on a carbon core. The FESEM and TEM images (Fig. S15) of hollow C@SiOC core-shell nanoparticles before B doping showed random packing with smooth surface. However, a partially interconnected assembly with highly rough surface was observed after B doping, proving the potential versatility of this BDIIA method.

Further, a detailed temperature-dependent investigation was conducted by changing the calcinating temperature to explore the formation kinetics of BDIIA (Fig. S16). It was found that as temperature increased at the same concentration of boric acid, the surface of the particles become rougher, attributed to enhanced kinetics at higher temperature. The high kinetics might also disrupt the dense stacking of nanoparticles, and thus weaken the connections between neighboring particles. The concentration of boric acid mainly tailored the etching depth of interphase, as more boric acid produced more boron oxide, which could melt more silicon oxide according to the principle of thermodynamics. A higher concentration of boric acid led to thicker etching depth and thus bigger connection. Accordingly, the size of the ‘neck’ connections and surface roughness could be well manipulated by simply tuning the calcination temperature or precursor/boric acid ratio.

One unique structural feature in the as-prepared B-SiOC assembly is the tough connection between primary nanospheres. Such connection configuration could substantially enhance both interfacial stability and electronic coupling between neighboring nanoparticles, which is desirable for energy storage applications [39,40]. To demonstrate this, B-SiOC assemblies were investigated on the half-cell configuration. The cyclic voltammetry (CV) curves of the B-SiOC-2 sample displayed the typical characteristics of silicon oxides (Fig. S17). Figure S18a shows the cycling performance of the four samples. Generally, all the electrodes manifested stable cycling performances, and the B-SiOC-2 possessed the highest specific capacity. Moreover, the B-SiOC-3 electrode also revealed high specific capacity and stable cycling (over 700 mAh g^−1^ remained after 300 cycles). However, the B-SiOC-1 electrode delivered relatively low charge/discharge capacity and capacity fading after 150 cycles, which could be attributed to the lower B doping content and incomplete assembly. Although B-SiOC-4 possessed ultra-stable cycling performance compared with other samples, inferior specific capacity was obtained, probably because of the poor electrical conductivity induced by lower carbon content. This can be supported by the Nyquist plot measured from electrochemical impedance spectroscopy (EIS), in which the semicircle refers to the charge-transfer resistance. As shown in Fig. S18b, the kinetic behavior of the four samples after 300 cycles displayed the same tendency. Both B-SiOC-3 and B-SiOC-2 electrodes showed small resistance because of their perfect assembly and thus interconnected carbon framework. However, the B-SiOC-1 and B-SiOC-4 anodes manifested larger charge-transfer impedance on account of partial assembly and inferior conductivity, respectively. The results were verified by the fitted data based on the corresponding equivalent electrical circuit, see the inset in Fig. S18b. The Re represents the ionic resistance of the electrolyte, CPE_sei_ and R_sei_ represent the capacitance and resistance SEI of (solid electrolyte interface) films, respectively. CPE_dl_, R_ct_ and Z_w_ represent the double layer capacitance, charge transfer resistance and Warburg impedance. The B-SiOC-2 electrode possesses resistance (R_ct_) of 149 Ω, the lowest among the four electrodes (Table S1), further indicating it has the highest charge transfer kinetics.

**Figure 5.**
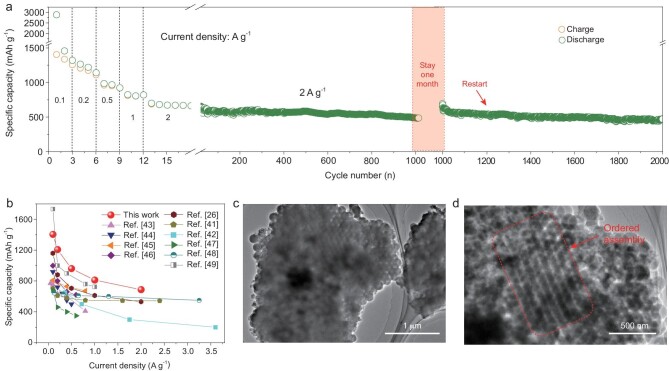
(a) Rate and cycling performance of B-SiOC-2 anode. (b) Rate performance comparison with SiOC-based or SiO_x_/C-based anode materials in the literature. (c, d) TEM images of BD-SiOC-2 electrode after cycling.

The rate capabilities of B-SiOC-2 anodes were investigated by increasing the test current density from 0.05 to 2 A g^−1^ (Fig. 5a). The reversible capacities reach 1404, 1207, 961, 812 and 688 mAh g^−1^ at current densities of 0.1, 0.2, 0.5, 1.0 and 2.0 A g^−1^, respectively. Impressively, this electrode undergoes negligible capacity fading after 1000 cycles at a high rate of 2 A g^−1^, displaying a reversible capacity of 485 mA h g^−1^ with a 0.03% decay per cycle. After one month, continued cycling at 2 A g^−1^ still resulted in an initial capacity of 644 mAh g^−1^, and capacity retention of over 72% can be achieved after the next 1000 cycles. Such rate performance and cycling stability outperform those of most SiOC-based [41,42] or SiO_x_/C-based [26,43–49] anodes reported previously (Fig. 5b). To investigate the structural stability of this assembly during cycling, ex situ SEM (Fig. S19) and TEM (Fig. 5c and d) studies were performed. Ordered assembly was observed (red-outlined area in Fig. 5d), with robust connections with each other, which confirms the highly interfacial stability of such assembly.

This superior battery performance of B-SiOC assemblies is ascribed to the unique structural characteristics. First, this space-efficient assembly of primary nanoparticles into a micrometer-scale secondary structure effectively reduces interfacial areas between electrodes and electrolyte, reducing the formation of SEI. Second, unlike traditional assembly in which primary particles experience weak connections, the robust connections between adjacent nanoparticles can significantly reduce the interphase resistance and thus provide a continuous electron pathway, which facilitates electronic connectivity within the electrode. To prove this, selective etching of silicon oxide in the B-SiOC-2 assembly was performed (Fig. S20). The remaining carbon nanospheres can maintain the micrometer-scale assembly, and most importantly, neighboring primary nanoparticles still maintain tough connections with each other, giving powerful proof to formation of a continuous electron network. In addition, doping of B into the carbon framework can enhance the adsorption with lithium ions, which is conducive to improvement of rate performance. The multiple merits give rise to a synergism that enables a constructed half-cell with excellent rate capacity and long cycle life.

## CONCLUSION

In summary, we have demonstrated, for the first time, an interconnected assembly of SiOC nanoparticles induced by B doping. A novel melt-etching and nucleation-growth mechanism was proposed through detailed analysis of the chemical bonding environment of B on the surface. Specifically, co-melting of boron oxide and SiO_x_ disrupts the solid interphase at elevated temperature, forming a moving layer composed of boron oxide, SiO_x_ and carbon domains. Dense packing of SiOC nanoparticles can then be induced on the guidance of this flowing interface layer, and meanwhile, various B doping structures are formed. During the cooling stage, ‘neck’-like connections between adjacent nanoparticles, along with surface roughening, are formed during the nucleation and growth process. The size of the ‘neck,’ which is controlled by the etching depth, and surface roughness, which is influenced by reaction kinetics, can be regulated by changing the content of boric acid added and reaction temperature, respectively. This uniquely robust coupling in the as-formed assemblies gives the manifested anode ultra-stable long-term life with high specific capacity at relatively high current density. This BDIIA approach is anticipated to be extended to prepare multifunctional materials such as B doped carbon assemblies after selective etching, which can be applied to various applications.

## METHODS

### Synthesis of B-SiOC assembly

First, boric acid and SiOC nanoparticles [28] (see Supplementary data) with different mass ratios were dispersed in deionized water under sonication for 60 min to obtain a uniform mixture. The mixture was freeze-dried and then carbonized at 900°C under an Ar/H_2_ atmosphere for 240 min at a heating rate of 5°C min^−1^. The carbonized sample was washed with deionized water three times and dried at 40°C.

### Preparation of comparative samples

Carbon nanospheres were obtained from carbonization of resorcinol/formaldehyde (RF) resin polymer spheres, which were synthesized according to a previous report [50]. The SiO_2_@carbon core-shell nanospheres were synthesized by a one-pot sol-gel approach [51]. Typically, 6.92 mL of tetraethyl orthosilicate (TEOS) were added in a solution containing 20 mL of deionized water, 140 mL of absolute ethanol and 6 mL of aqueous ammonia. After 15 min, 0.8 g of resorcinol and 1.12 mL of formaldehyde (37 wt%) were added to the solution and the mixture was stirred for 24 h. After centrifugation and drying at 60°C, the as-prepared samples were carbonized at 800°C. The synthesis procedure for SiO_2_ and another SiOC nanosphere are the same with the SiOC nanoparticles (see Supplementary data), except that the precursors were TEOS and 1,2-bis(triethoxysilyl) ethenylene (BTEE), respectively, instead of BTEB. The hollow carbon@SiOC core-shell nanospheres were synthesized by a sol-gel method and subsequent carbonization. Typically, 0.1 g of resin polymer spheres were added in a mixture containing 120 mL of deionized water, 15 mL of absolute ethanol and 1 mL of aqueous ammonia. After stirring for 30 min, 0.2 mL of BTEB were added to the solution and the mixture was further stirred for 12 h. After centrifugation and drying at 60°C, the as-prepared samples were carbonized at 800°C.

## Supplementary Material

nwaa152_Supplemental_File
