# Supplementary material for: Boron doping-induced interconnected assembly approach for mesoporous silicon oxycarbide architecture
Source: Natl Sci Rev. 2020 Jul 2;8(6):nwaa152. doi: 10.1093/nsr/nwaa152 (PMC8288169; doi:10.1093/nsr/nwaa152)
Supplement: nwaa152_Supplemental_File [file nwaa152_supplemental_file.docx]

**Supplementary data for**

**Boron Doping-Induced Interconnected Assembly Approach for Mesoporous Silicon Oxycarbide Architecture**

Guanjia Zhu^1^, Rui Guo^1^, Wei Luo^1^, Hua Kun Liu^2^, Wan Jiang^1^, Shi Xue Dou^2^ and Jianping Yang^1,^*

^1^State Key Laboratory for Modification of Chemical Fibers and Polymer Materials, International Joint Laboratory for Advanced Fiber and Low-dimension Materials, College of Materials Science and Engineering, Donghua University, Shanghai 201620, P. R. China

^2^Institute for Superconducting & Electronic Materials, Australian Institute of Innovative Materials, University of Wollongong, Innovation Campus, Squires Way, North Wollongong, NSW 2500, Australia

**^*^**Corresponding Author.

^*^Email: [jianpingyang@dhu.edu.cn](mailto:jianpingyang@dhu.edu.cn)

**Methods**

**Synthesis of SiOC nanoparticles.** SiOC nanoparticles were prepared as previously reported. In a typical procedure, 800 mg of hexadecyl trimethyl ammonium bromide (CTAB) were dispersed in a solution containing 280 mL of deionized water and 120 mL of absolute ethanol. Then 4 mL of aqueous ammonia (NH_4_OH, 25 wt %) was added. After stirring for 1h, 1 mL of 1,4-bis(triethoxysilyl)benzene (BTEB) was added dropwise to the dispersion under continuous stirring for 12 h. The monodispersed nanospheres could be obtained after centrifugation and drying at 60 °C. The as-synthesized nanospheres were heated in Ar at 800 °C for 8 h with a temperature ramping rate of 2 °C/min.

**Characterization**

Field emission scanning electron microscopy (FESEM) images were obtained on a Hitachi SU8010 (Japan) field-emission SEM. Transmission electron microscopy (TEM) measurements were conducted on a JEM-2100 F microscope (JEOL, Japan) operated at 200 kV. Field emission transmission electron microscopy (FETEM) were conducted on a Talos F200S microscope operated at 200 kV. Inductive coupled plasma-atomic emission spectrometer (Prodigy-ICP) was performed to determine the relative boron contents to silicon. In total, 10 mg of the samples was completely dissolved into 5 mL concentrated nitric acid and a few drops of hydrofluoric acid using a sonication bath for 60 min. Dissolving-time-dependent ICP experiments were carried out by withdrawing 500 µL of the solution at time 10 s, 30 s, 1 min, 2 min, 5 min, 30 min and 60min. Wide-angle X-ray diffraction (XRD) patterns were detected on a Rigaku D/Max-2550 PC diffractometer (Tokyo, Japan) for 2θ ranging from 10° to 90°. Nitrogen sorption isotherms were measured at Autosorb iQ machine. Before measurements, the samples were degassed in a vacuum at 200 °C for at least 10 h. The Brunauer-Emmett-Teller (BET) method was utilized to calculate the specific surface areas using the adsorption data at P/P_0_ = 0.02 - 0.20. The pore size distribution was calculated from the adsorption branch by using the DFT model. The total pore volume (V_total_) was estimated from the adsorbed amount at P/P_0_ = 0.995. Raman spectra were obtained with a Dilor LabRam-1B microscopic Raman spectrometer (France), using a He-Nelaser with an excitation wavelength of 632.8 nm. Thermal gravimetric analysis (TGA) was carried out on a TG 209F1 apparatus under air atmosphere in the temperature range of 50-900 °C with heating rate of 5 °C /min.

**Electrochemical measurements.** The working electrode was prepared by mixing the active materials (70 wt %), super P (20 wt %), and sodium alginate (10 wt %) in deionized water. The obtained slurry was uniformed casted on copper foil and dried in a vacuum oven overnight. The mass loading of the active material was 1.0-1.5 mg cm^-2^. CR2032 coin cells with Li foil as the counter electrode, which were used to investigate the electrochemical performance, were assembled in an argon-filled glove box with tiny (<0.1 ppm) H_2_O and O_2_ contents. The electrolyte was 1.0 M LiPF_6_ in 3:4:3 (weight ratio) of ethylene carbonate / dimethyl carbonate/ diethylene carbonate, with 5 wt % fluoroethylene carbonate additive. Galvanostatic discharge-charge (GCD) experiments were performed on a New Battery Measurement System at various current rates in a cut-off voltage range between 0.01 and 3.0 V. Cyclic voltammety (CV) and electrochemical impedance spectroscopy (EIS) measurements were conducted using an electrochemical workstation (VMP3). The CV scanning rate was 0.1 mV s^-1^. Electrochemical impedance spectroscopy (EIS) was performed in a frequency range of 0.01-100k Hz and a potential amplitude of 5mV. All the electrochemical measurements were conducted at 25 °C.


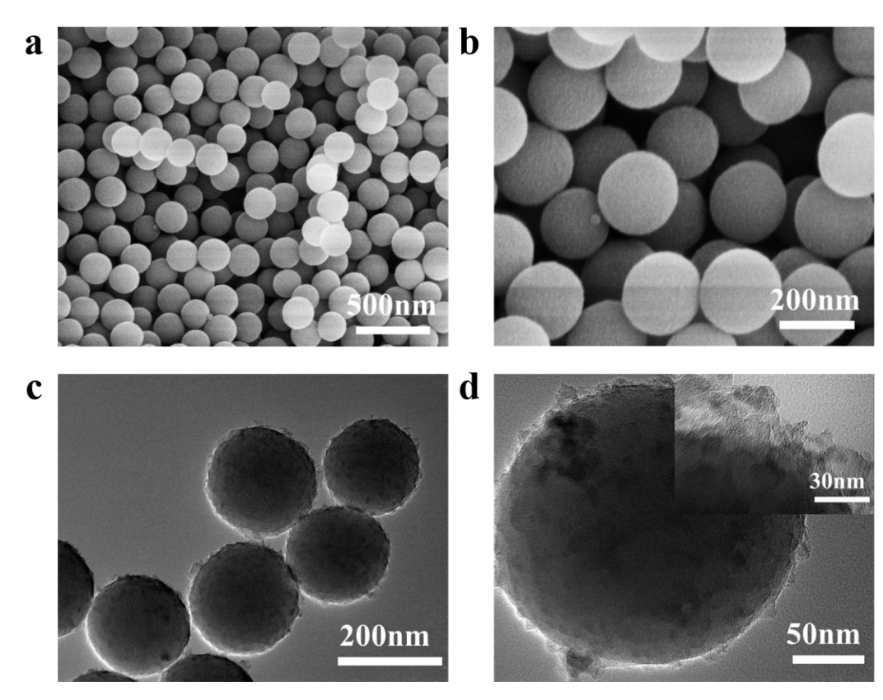


**Figure S1.** FESEM images (a, b) and TEM images (c, d) of B-SiOC-3 particles before calcination. Inset in d) is the magnified image of the shell of one B-SiOC-3 particle.


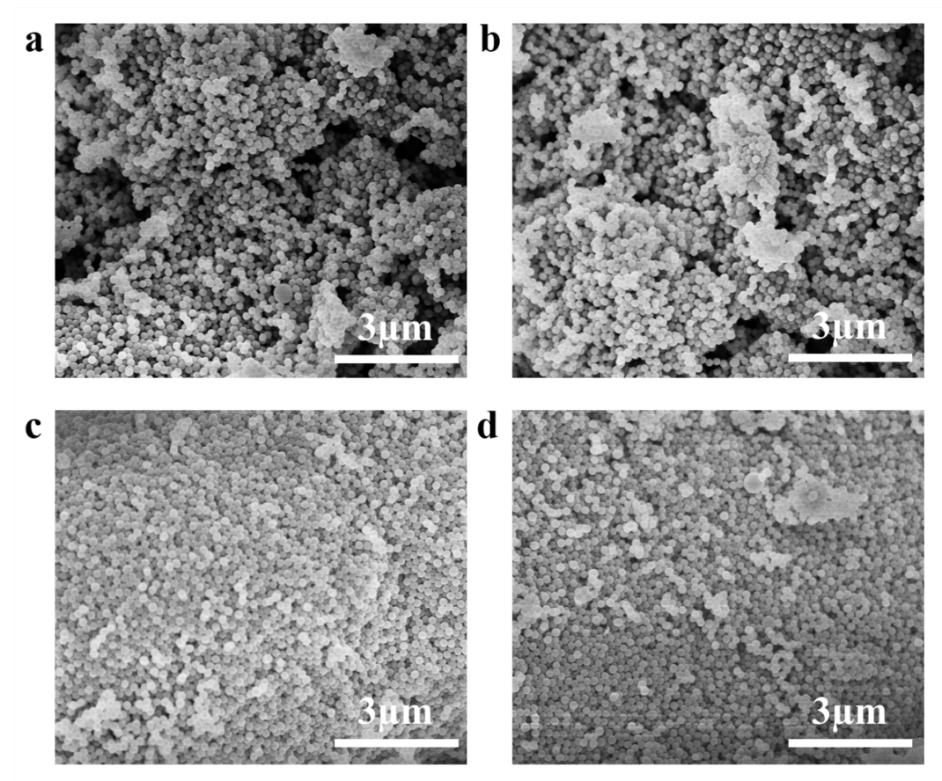


**Figure S2.** FESEM images of B-SiOC-1 (a), B-SiOC-2 (b), B-SiOC-3 (c) and B-SiOC-4 (d) before calcination.


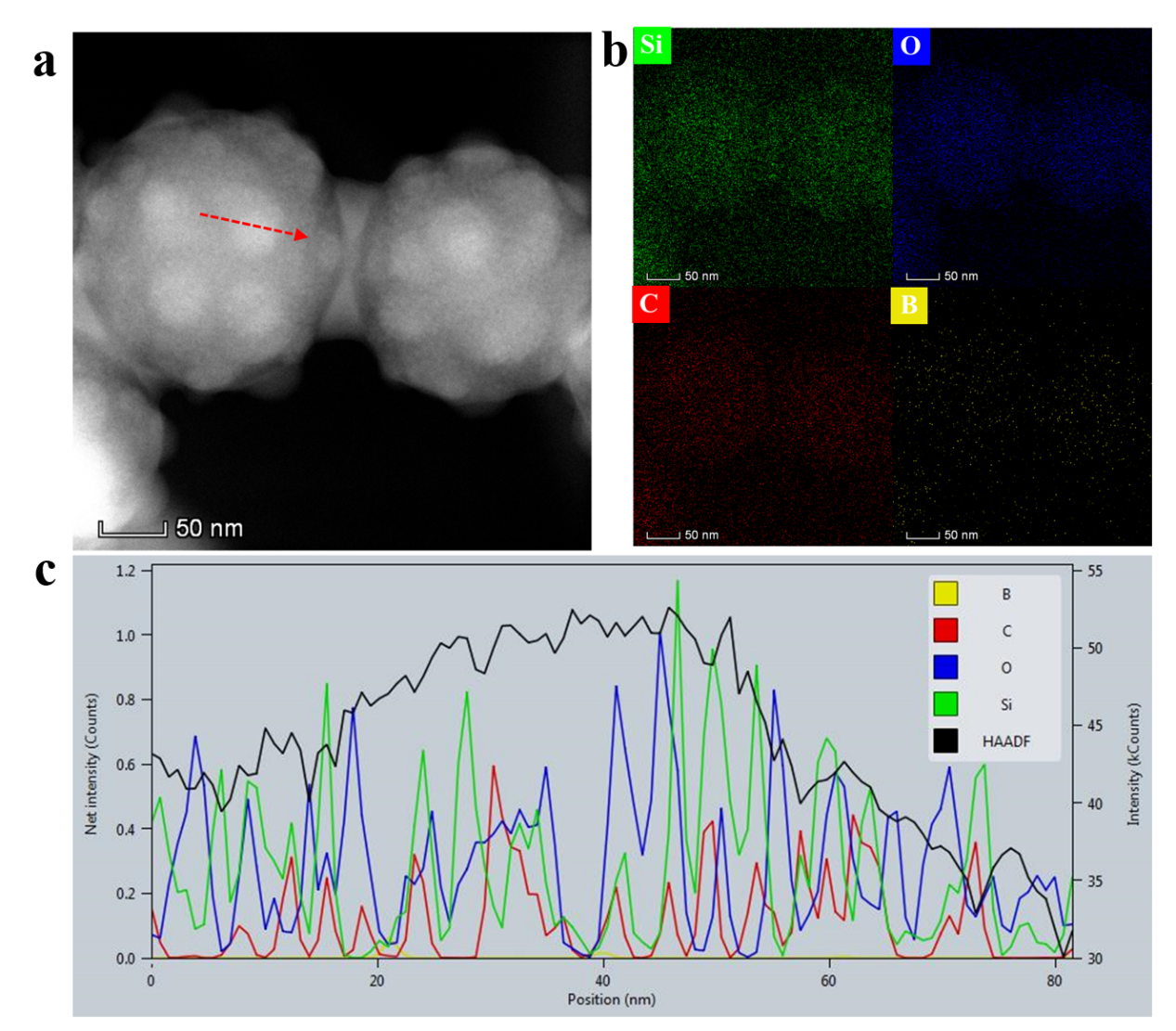


**Figure S3.** a) Dark-field TEM image and b) elemental mapping of the B-SiOC-4 assembly. c) EDS line mapping profiles along the direction indicated by the red arrow in (a).


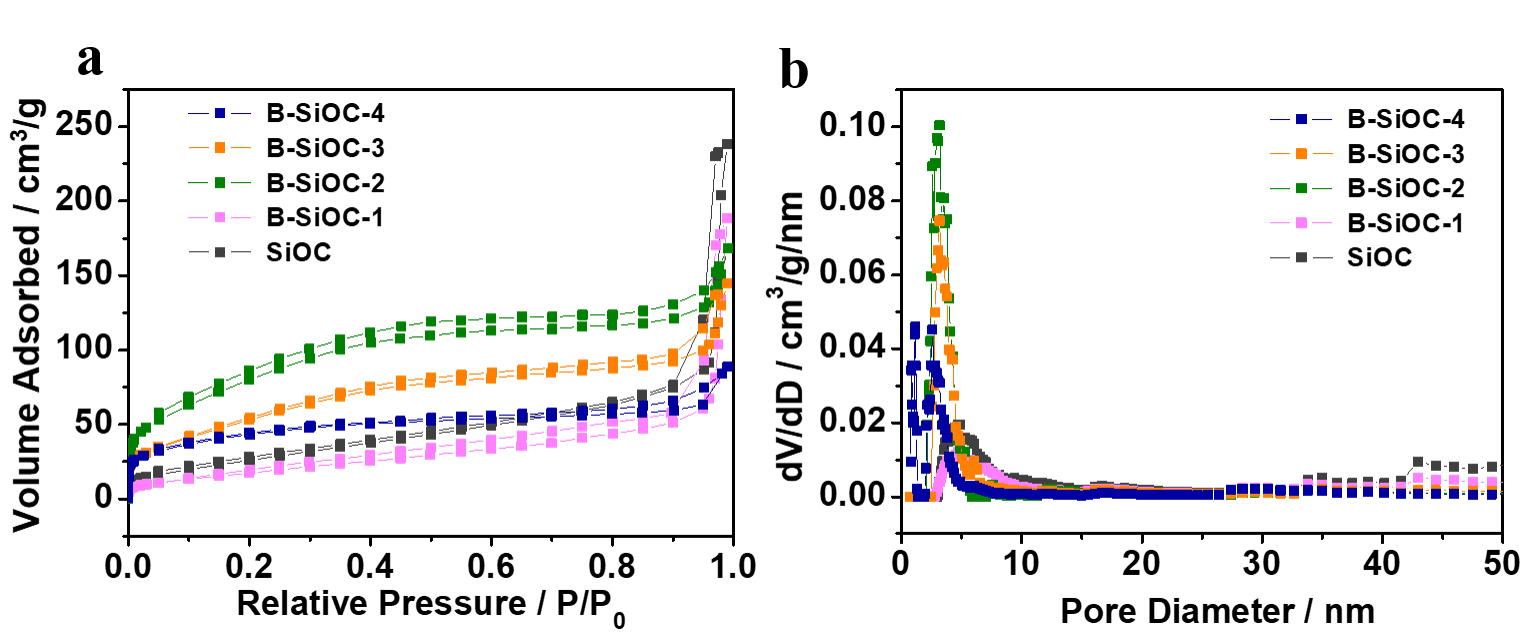


**Figure S4.** A nitrogen adsorption-desorption isotherm of B-SiOC samples (a) and the corresponding pore size distribution profile (b).


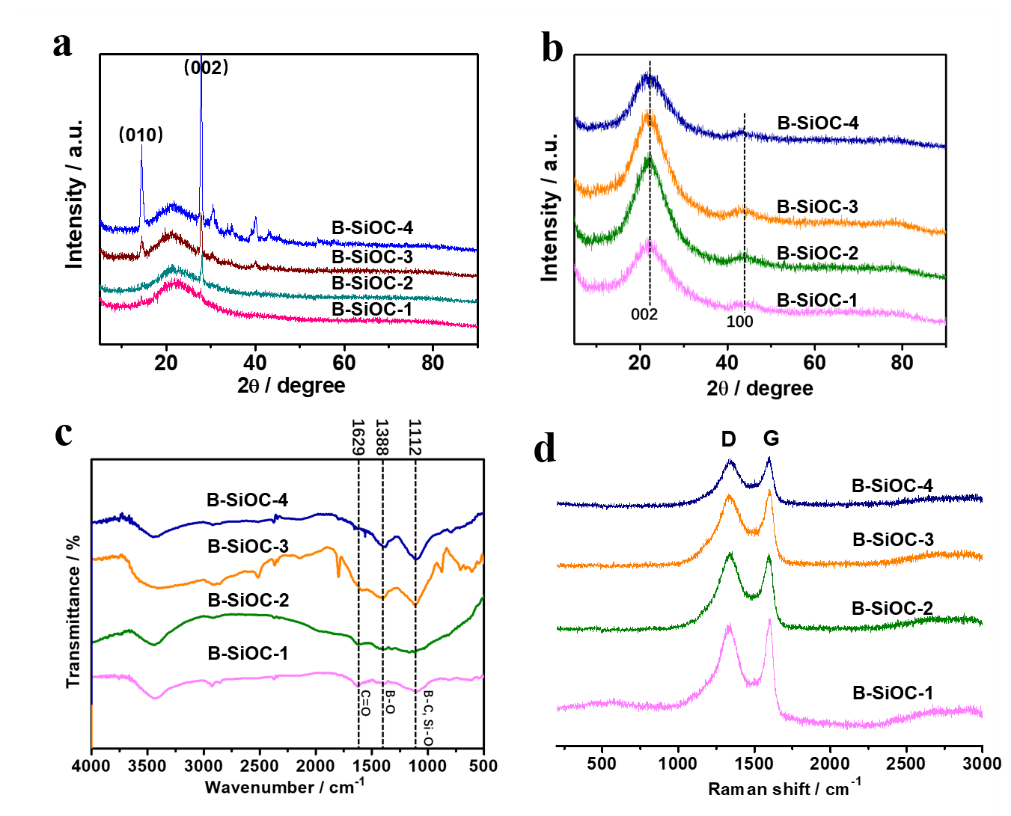


**Figure S5.** XRD patterns of B-SiOC samples before calcination (a) and after calcination (b). Raman shift (c) and FT-IR spectra (d) of B-SiOC after calcination.


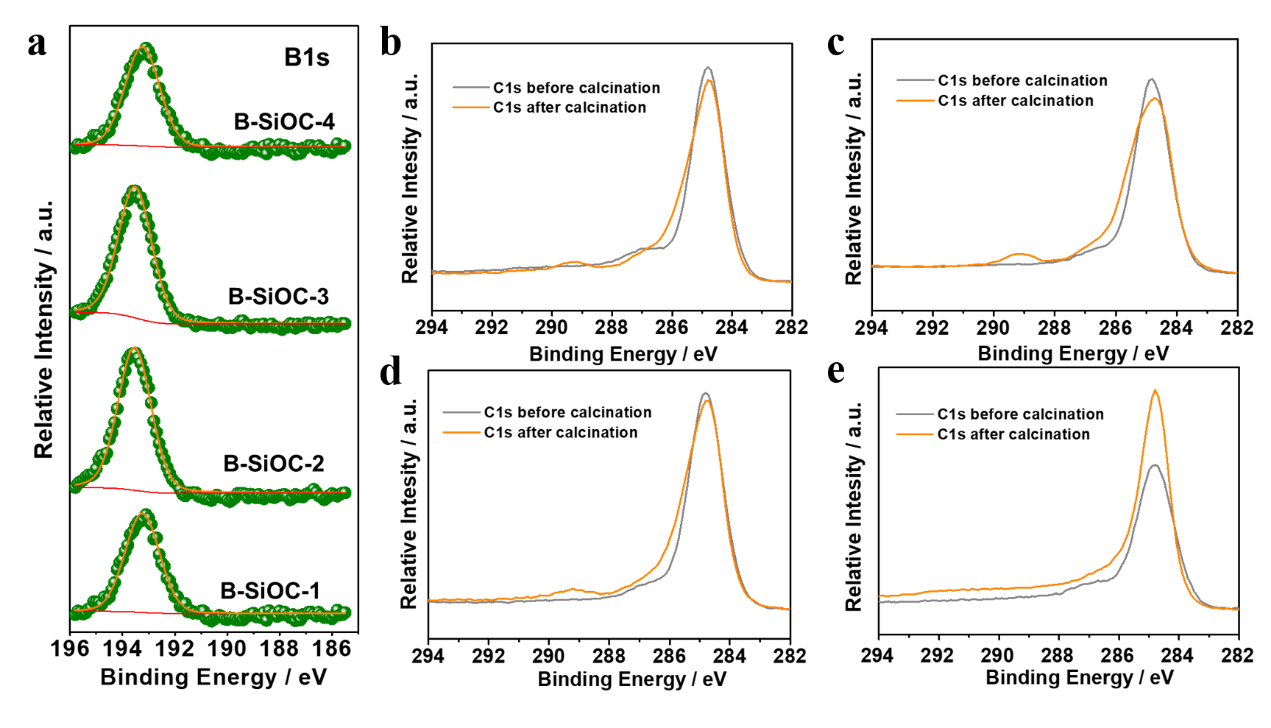


**Figure S6.** B1s region (a) of the high-resolution XPS spectrum of B-SiOC particles before calcination. C 1s region of the high-resolution XPS spectrum of B-SiOC-1 (b), B-SiOC-2 (c), B-SiOC-3 (d) and B-SiOC-4 (e).


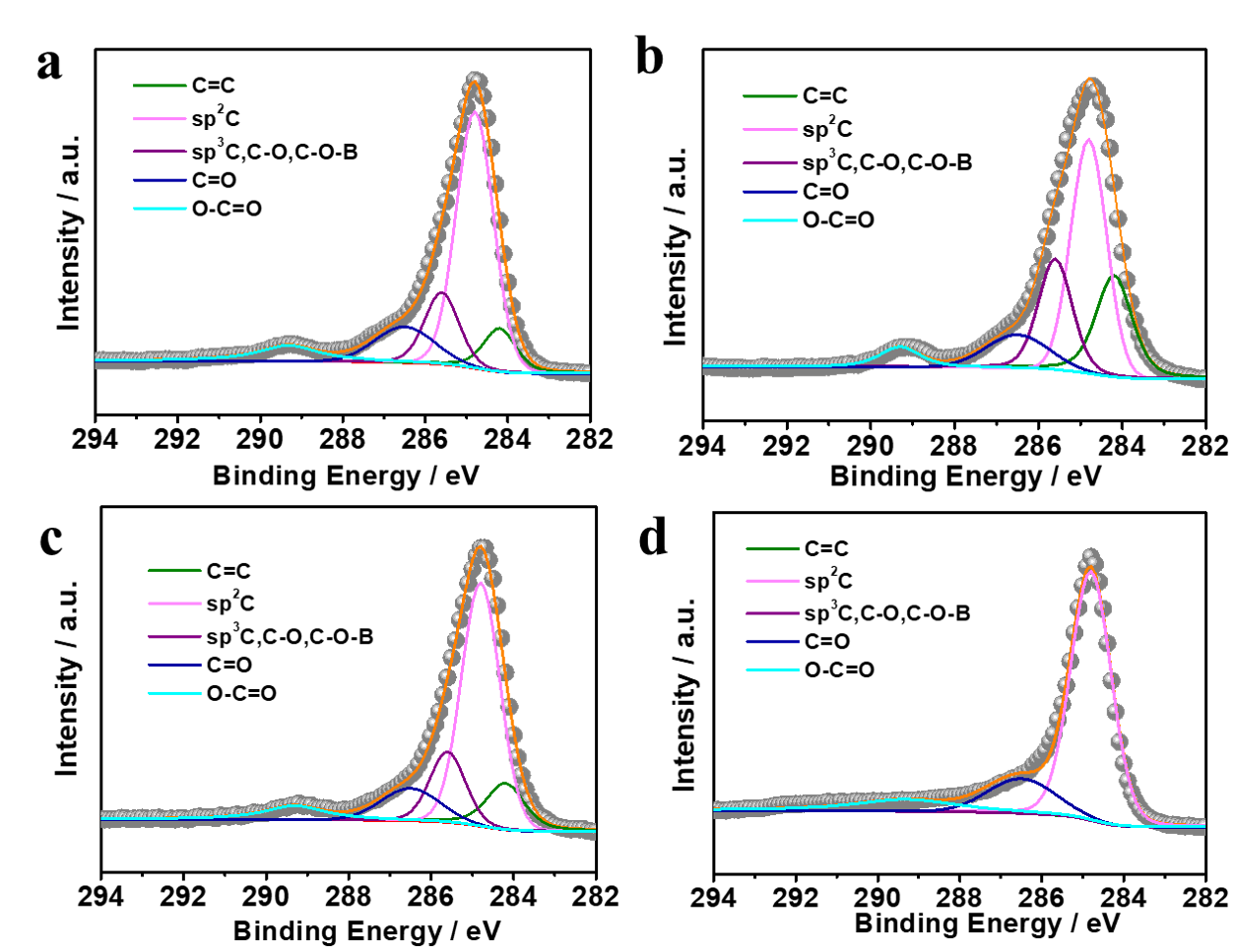


**Figure S7.** Deconvoluted C 1s spectrum of B-SiOC-1 (a), B-SiOC-2 (b), B-SiOC-3 (c) and B-SiOC-4 (d) after calcination.


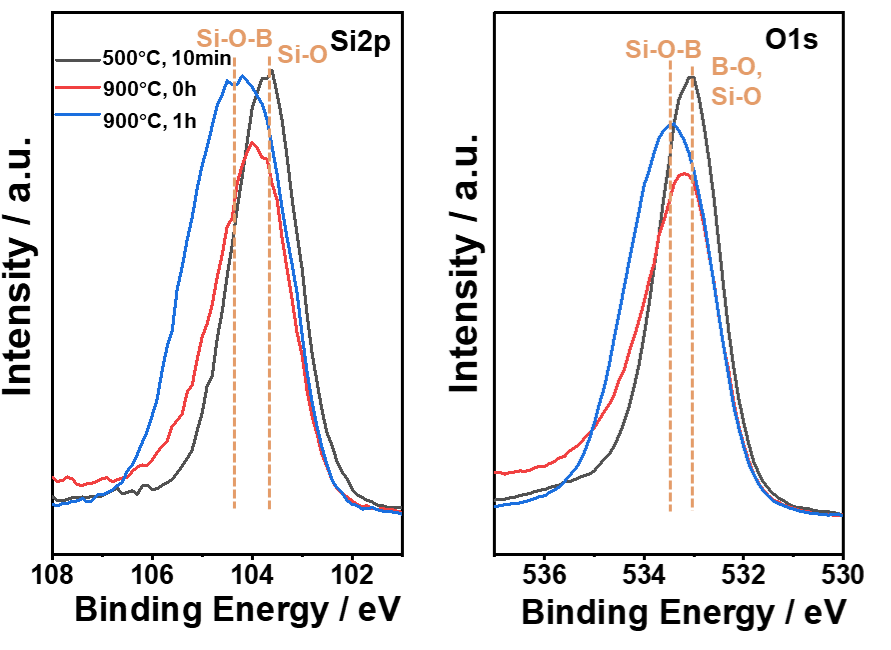


**Figure S8.** Si2p and O1s regions of the high-resolution XPS spectrum of the B-SiOC-2 at different reaction stages.


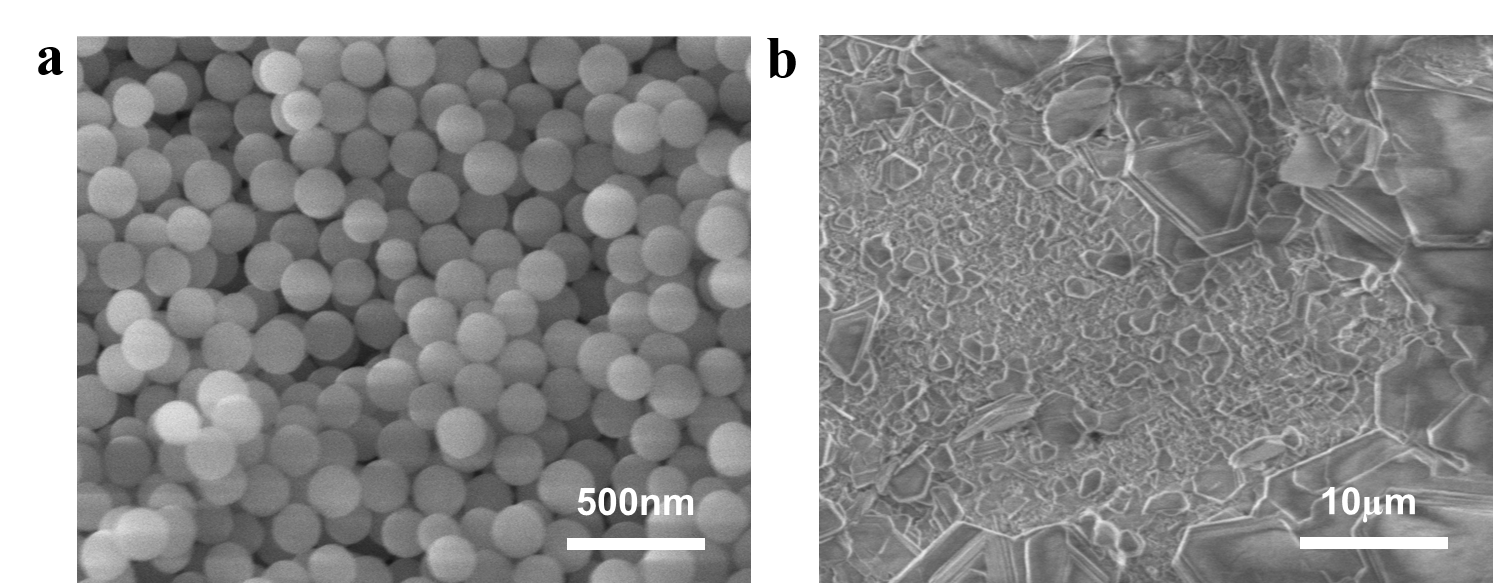


**Figure S9.** FESEM images of SiO_2_ nanoparticles before doping (a) and after doping (b).


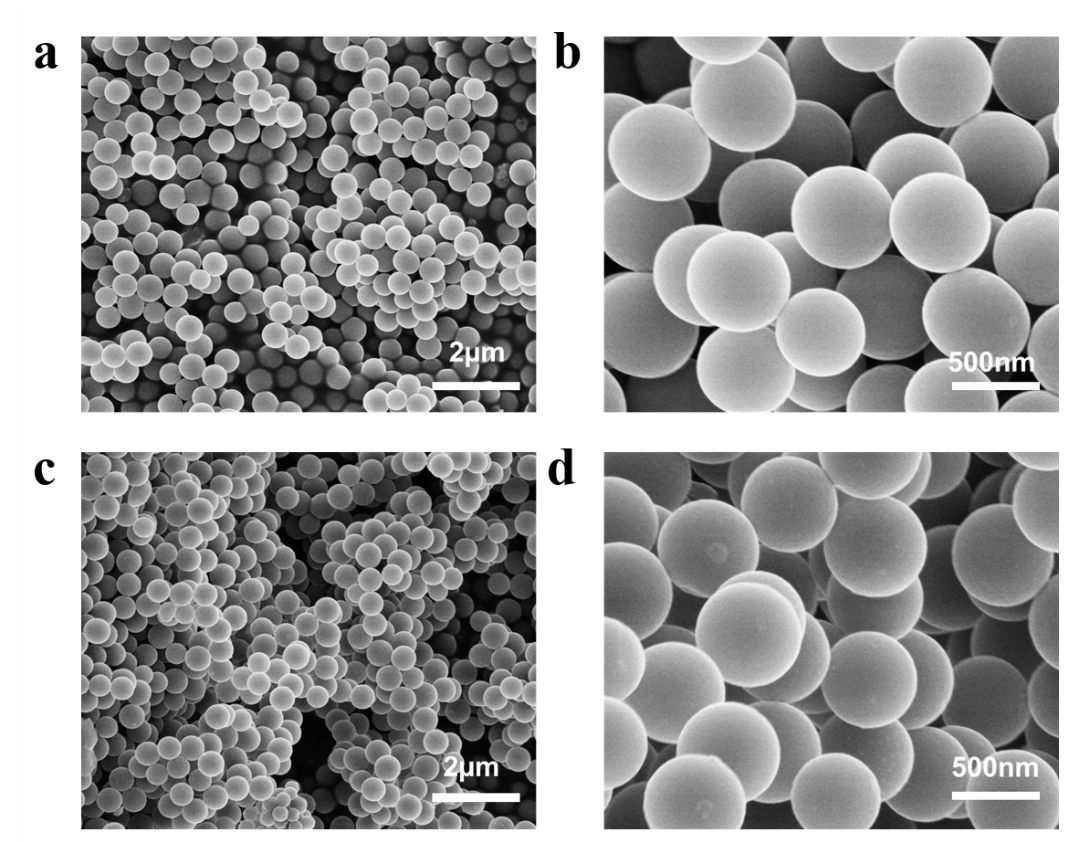


**Figure S10.** FESEM images of carbon nanoparticles before doping (a, b) and after doping (c, d).


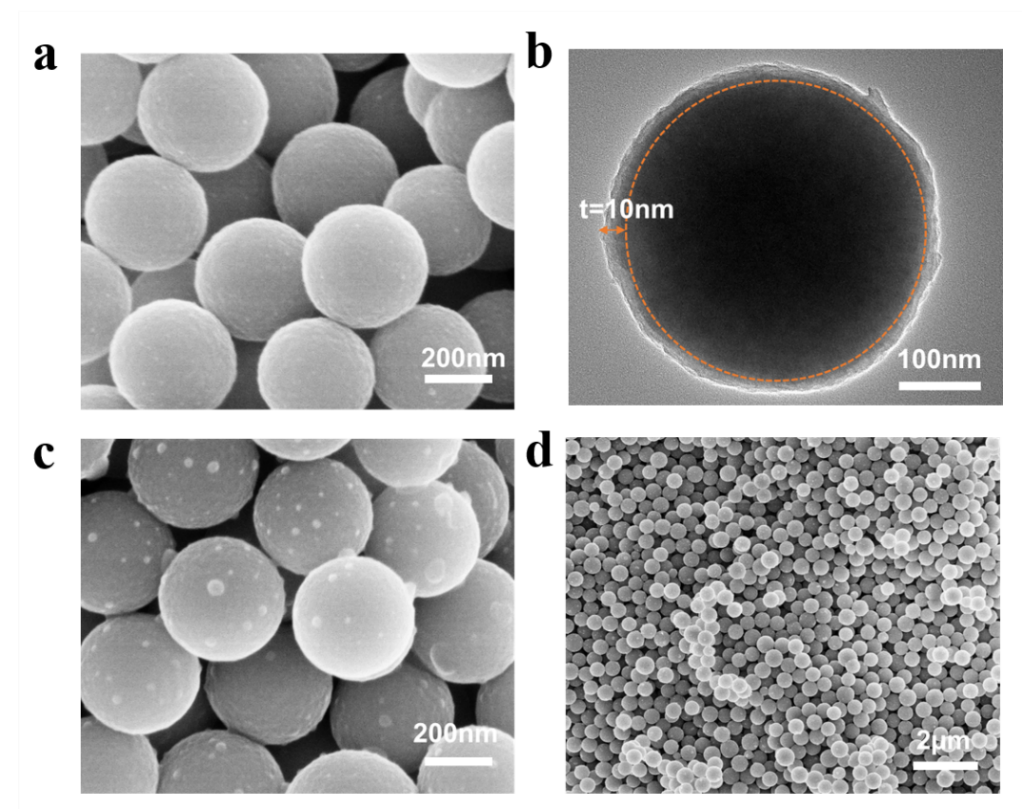


**Figure S11.** FESEM (a) and TEM (b) images of SiO_2_@carbon core-shell structures. FESEM images of the corresponding doping results (c, d).


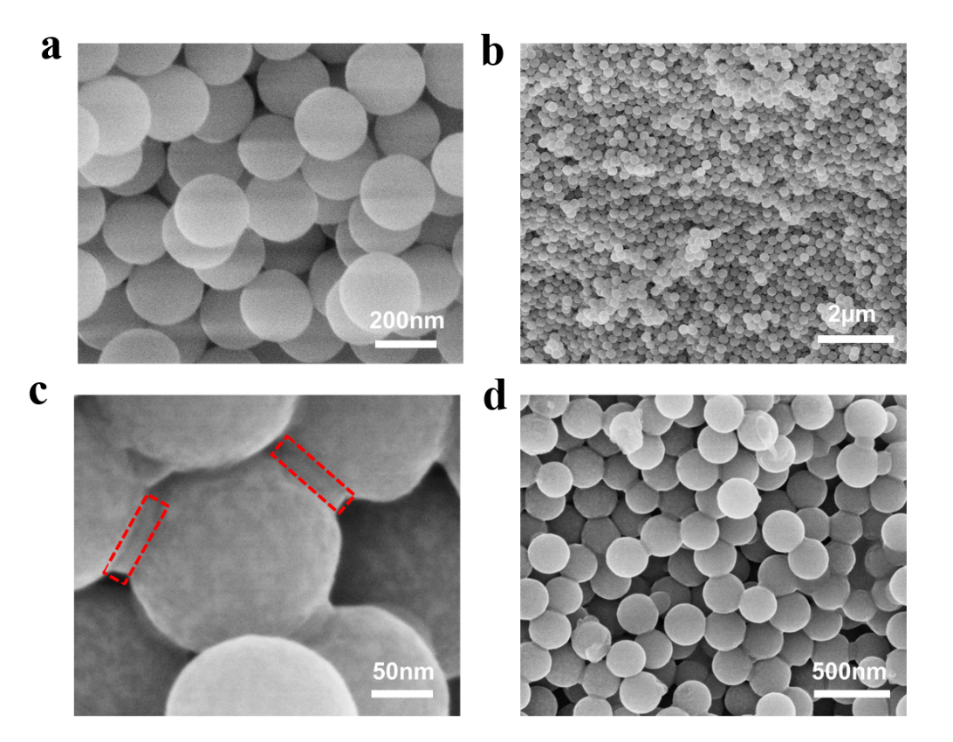


**Figure S12.** FESEM images of another SiOC nanoparticle derived from BTEE before doping (a, b) and after doping (c, d).


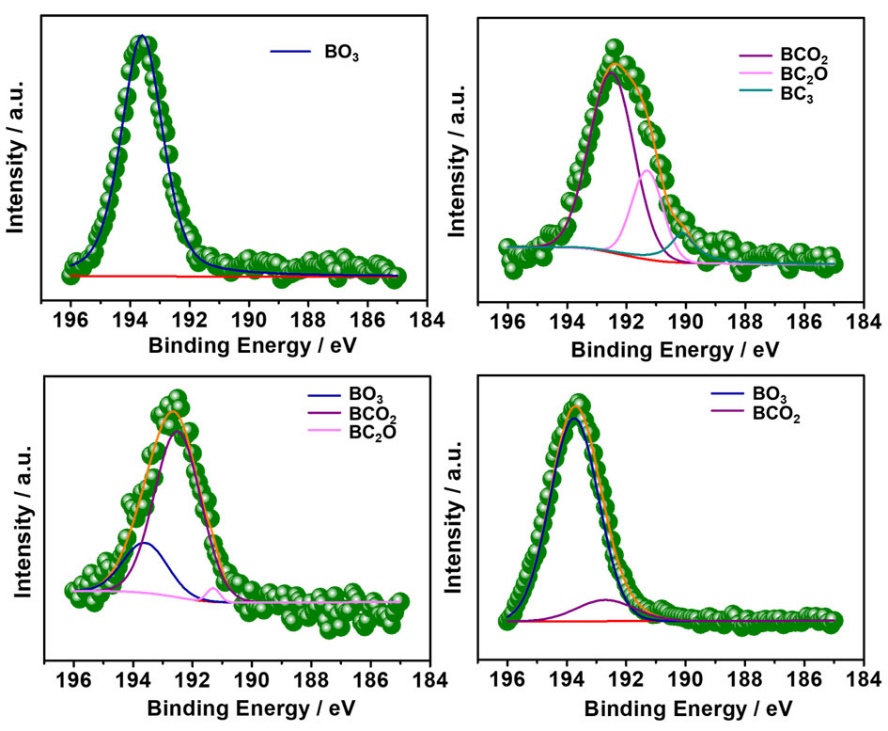


**Figure S13.** B1s regions of the high-resolution XPS spectrum of the SiO_2_ (a), carbon nanosphere (b), SiO_2_@carbon core-shell structures (c), and SiOC nanoparticle derived from BTEE.


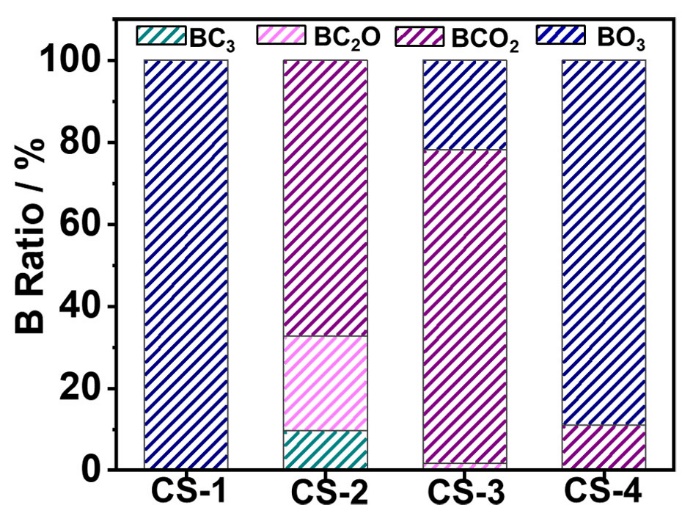


**Figure S14.** Percentages of different B types in the comparative samples (CS). CS-1 refers to SiO_2_, CS-2 is carbon nanosphere, CS-3 is SiO_2_@carbon core-shell structure, and CS-4 stands for SiOC nanosphere derived from BTEE.


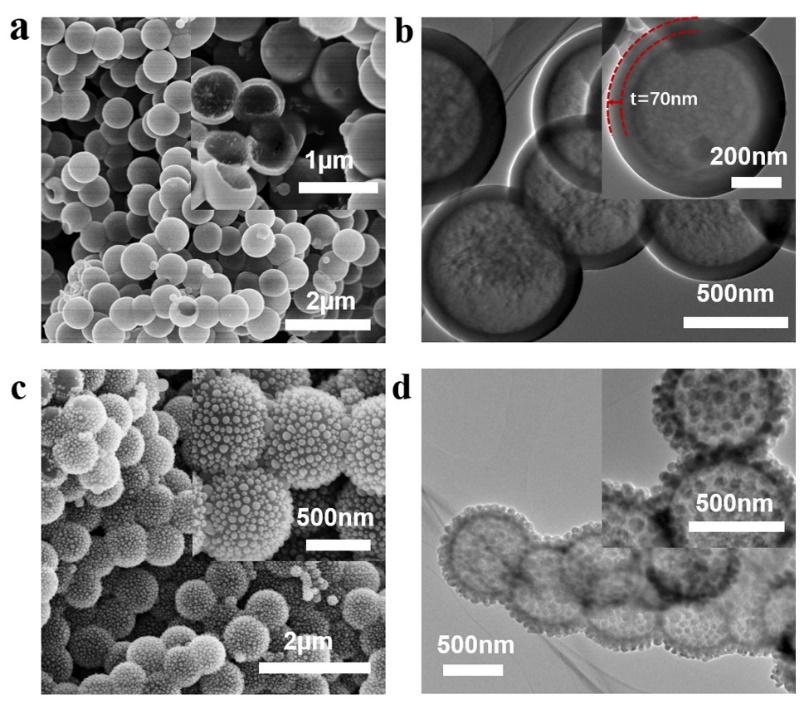


**Figure S15.** FESEM (a) and TEM (b) images of carbon@PMO hollow core-shell structures. FESEM (c) and TEM (d) images of the corresponding doping results.


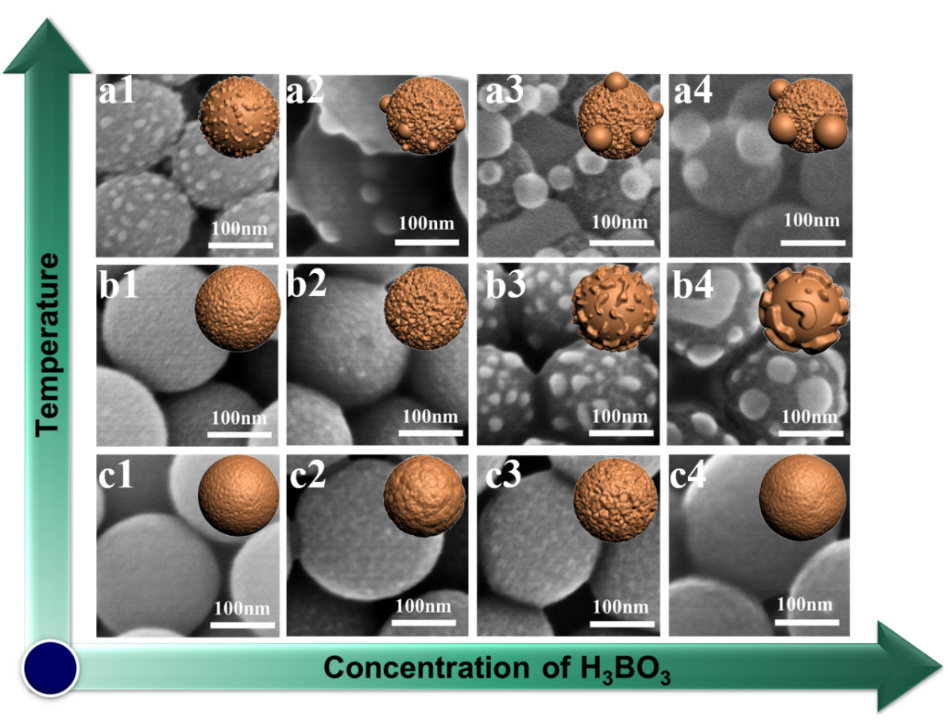


**Figure S16.** Temperature and boric acid concentration dependent FESEM images of B-SiOC assemblies. a-c refers to different calcination temperature (a is 950°C, b is 900°C, and c is 850°C). 1-4 represents different ratios between boric acid and precursors. The ratios are 1:10, 3:10, 5:10, and 10:10 respectively from 1 to 4.


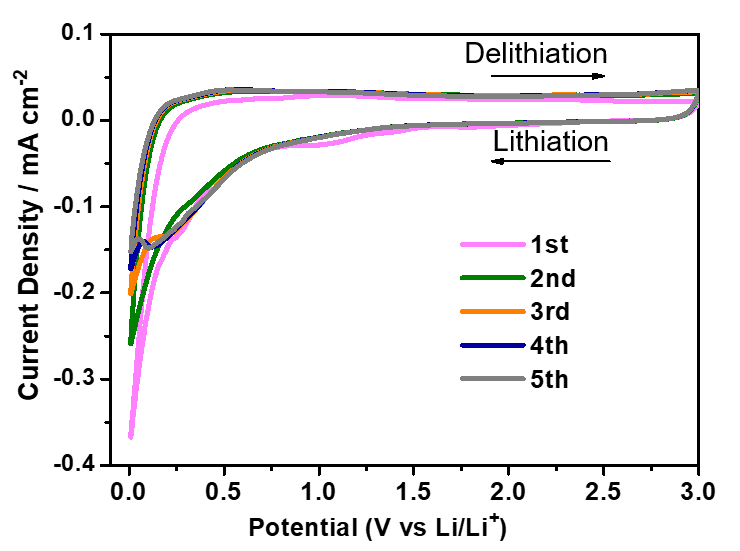


**Figure S17.** Cyclic voltammetry (CV) curves of B-SiOC-2 at a scanning rate of 0.1 mVs^-1^. In the first cycle, two reduction peaks can be obviously discerned. The peak at 1.04 V was relevant to the irreversible lithiation reaction of silicon oxide. The sharp peak below 0.25 V was ascribed to the alloying reaction between Si and Li. The first peak disappeared from the second cycle, which indicated the irreversibility of the corresponding reactions. While the cathodic peak below 0.25 V appeared in the subsequent cycles, suggesting the alloying reaction between Si and Li is reversible.


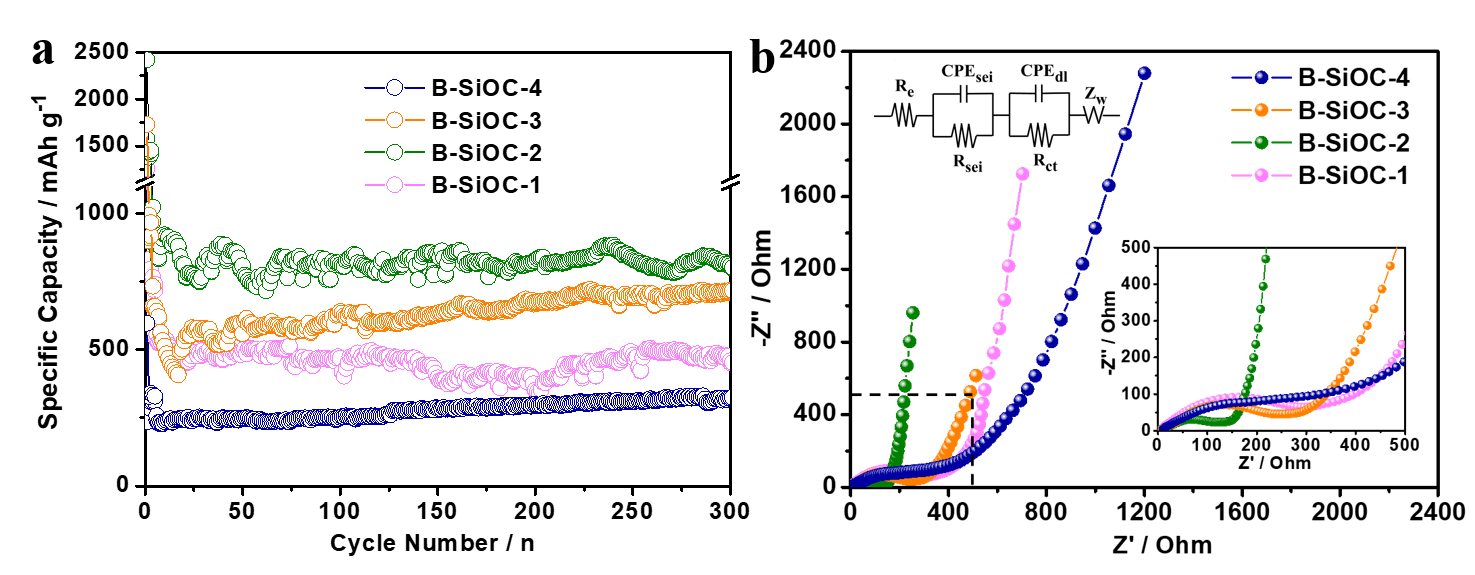


**Figure S18.** (a) Cycling performance of the four B-SiOC samples at 0.5 A g^-1^ (0.1 A g^-1^ for the first three cycles to activation). (b) electrochemical impedance spectroscopy (EIS) of the four B-SiOC samples after 300 cycles at 0.5 A g^-1^(inset in the upper left corner is the corresponding equivalent electrical circuit).


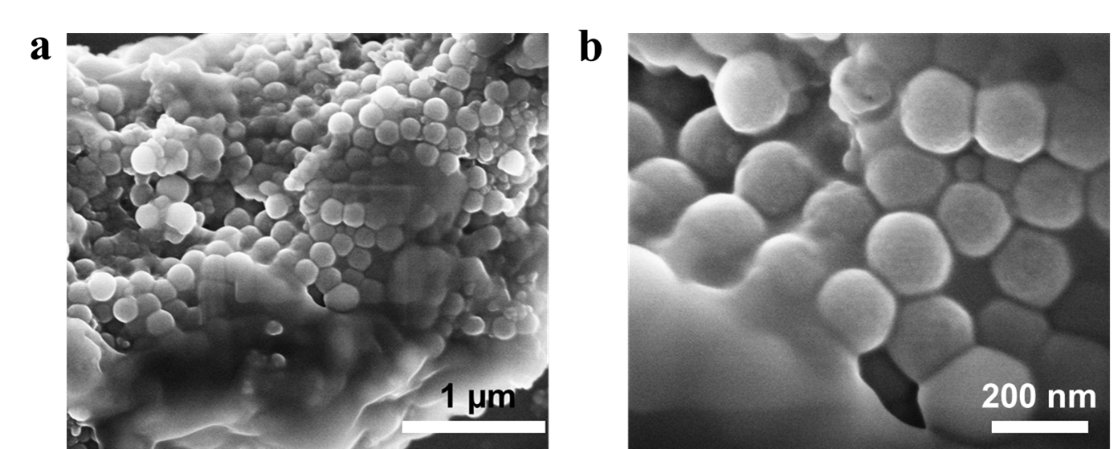


**Figure S19.** FESEM images of BD-SiOC-2 electrode after 100 cycles.


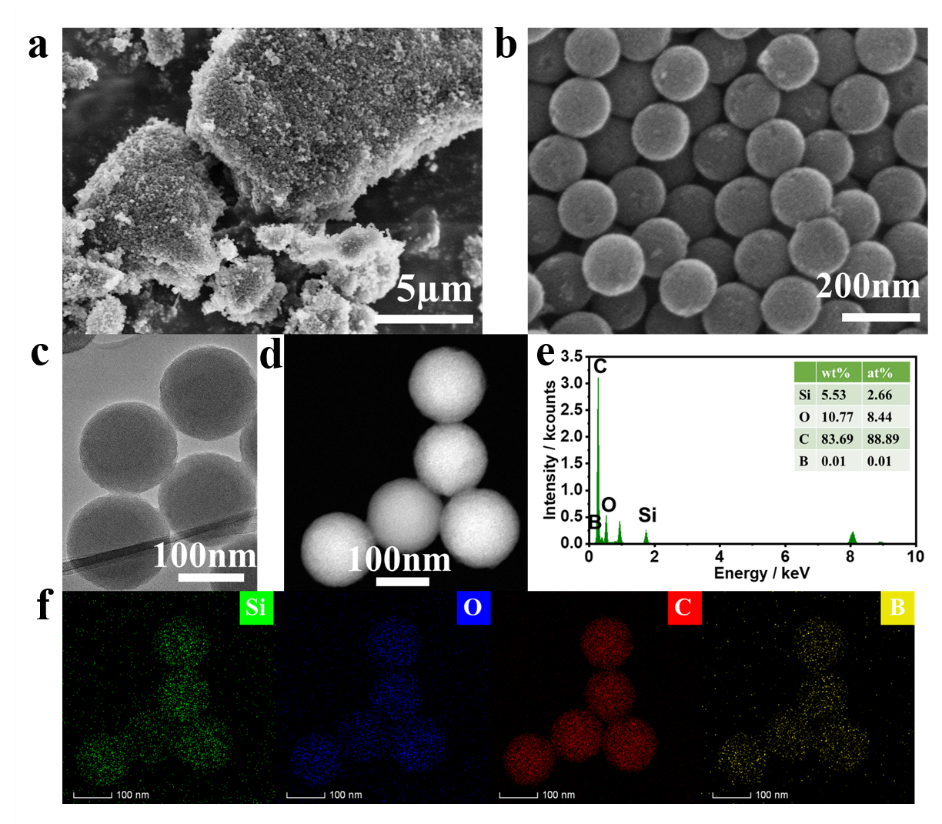


**Figure S20.** FESEM (a, b) and TEM (c) images of BD-SiOC-2 sample after HF etching. d) Dark-field TEM image, e) EDS elemental composition and f) corresponding elemental mapping of the sample.

**Table S1.** The fitted data of the four B-SiOC electrodes based on equivalent circuit.

| Samples | R_s_ | CPE_sei_ | R_sei_ | CPE_dl_ | R_ct_ | Z_w_ |
| --- | --- | --- | --- | --- | --- | --- |
| B-SiOC-1 | 4.117 | 0.01349 | 5.92E+10 | 6.52E-05 | 332 | 0.005809 |
| B-SiOC-2 | 3.905 | 0.01844 | 2.21E+04 | 3.97E-04 | 149 | 0.02896 |
| B-SiOC-3 | 5.825 | 0.03858 | 5.39E+03 | 2.04E-04 | 287 | 0.01407 |
| B-SiOC-4 | 4.862 | 0.01194 | 4.40E+10 | 1.92E-04 | 380 | 0.00316 |
